# 

**Published:** 1887-01-22

**Authors:** 


### The Hospital.

**An Institution, Family, and Parochial Journal of Hospitals, Asylums, and all Agencies for the Care of the Sick, Criticism and News.**

SATURDAY, JANUARY 22ND, 1887.

### The Literary Prize.

A PRIZE of **Two Guineas** will be given every month for the best story or incident based upon hospital, or asylum, or student life, or any incident connected with the professions of medicine or nursing.

### THE PRIZE COMPETITOR.

AT the request of many of our readers, we have determined to institute, in lieu of THE HOSPITAL "Charity Box," a

**Quarterly Prize Competition.**

We shall set each week two competitions, the nature of which we propose to vary from week to week, and we shall award at the end of each quarter, a

**Prize of Three Guineas**

to the Competitor who shall have sent in the largest number of correct or best answers to the PRIZE COMPETITOR. No one will be permitted to win the Prize twice in any one year, but we shall award annually an additional

**Prize of Five Guineas**

to the competitor who shall have sent in the largest number of correct or best answers during the twelve months. We hope this feature of THE HOSPITAL will prove one of interest and amusement to our numerous readers, and that it will be very generally participated in.

**No. 1. Double Acrostic.**

Lo, England's *patron saint* of glorious fame,
Links with my *hospital* his mighty name.
Caring for *self*, but nobody more,
Loving my ego the wide world o'er.
Holding in Spain the *highest command*
Of the *army* throughout the whole land.
Common to men, to wheat, and a wall,
But donkeys have the *longest of all*.
Right after my *owner* comes my *ship*,
(The order reversed may sometimes slip).
Among the *Jews* I'm often teaching
The law and all that's learned preaching.
I am just the opposite of *small*;
Tacked to Peter; not annexed to Paul.
A *burning cone* 'neath Italy's skies,
Spreads grave terror when its ashes rise.
Out of me, tales you *ne'er should tell*,
Boys and girls oft like me none too well.

**No. 2. A Hospital Appeal.**

Write, in prose or verse (not exceeding one hundred words) an APPEAL on behalf of A General Hospital. Credit will be given for answers which are epigrammatic, apposite and pithy. The briefer the APPEAL, the better.

Answers must be addressed to THE PRIZE COMPETITOR, THE HOSPITAL, Norfolk House, Norfolk Street, Strand, W.C., not later than Saturday, 29th inst. The full name and address must in all cases be given, but a *nom de plume* may be added for publication. Only one side of the paper should be written on. The "Prize Competitor" Coupon (see advertisement pages) must be enclosed with each reply.

### THE CHILDREN'S CORNER.

### OUR PASTIMES.

**Children's Quarterly Prize Competition.**

| | |
|---|---|
| Began .. .. .. .. .. | 1st Jan., 1887 |
| Ends .. .. .. .. .. | 26th Mar., 1887 |

PRIZES.

| | |
|---|---|
| 1st .. .. .. .. .. | Thirty Shillings |
| 2nd .. .. .. .. .. | Twenty " |
| 3rd .. .. .. .. .. | Ten " |
| 4th .. .. .. .. .. | Five " |

will be awarded to the four competitors who shall have sent in, during the thirteen weeks, the largest number of correct answers to our pastimes.

**Special Letter Prize.**

Each competitor is invited to forward a little letter, with the answers, addressed to the Puzzle Editor. The letters sent by competitors may be upon any subject they choose, such as their studies, their little pets, their little brothers, sisters or companions, little stories or recollections about the hospitals, or anything else they please. The letters should *not be too long*, and attention should be paid to the handwriting and the grammar. Only one side of the paper should be written on. The Puzzle Editor has decided to award a SPECIAL LETTER PRIZE OF TEN SHILLINGS for the best letters contributed during each Quarterly Competition.

**Fourth Competition.**

No. 23. CONUNDRUM. Why is Richmond like the letter R ?

No. 24. HIDDEN PROVERB. Supply the missing letters in the following: S t c h n t m s v s n n.

No. 25. CONUNDRUM. A merchant has two addresses ; one in the City where he conducts his business, and the other in a western suburb, where he resides. Why should his friends be able to remember his City address better than the other one ?

No. 26. SQUARE WORD. A girl's Christian name ; a word meaning lawful ; one who acts for another ; to set in order ; to change or make different.

No. 27. A CROSS IN A DIAMOND. An aspirate ; a negative ; a word meaning to make haste ; a word meaning to make stupid or insensible ; one who professes the art of healing ; a generic name for creeping animals ; a piece of level country ; everyone ; a letter that frequently forms a plural.

```
        *
      · * ·
    · · * · ·
  · · · * · · ·
* * * * * * * * *
  · · · * · · ·
    · · * · ·
      · * ·
        *
```

**Results of Second Competition.**

No. 8. MISSING VOWEL PUZZLE. This was a very easy one : "A bird in the hand is worth two in the bush."

No. 9. CONUNDRUM. Why are a little girl's frocks which are quite worn out like children whose parents are dead? Why, because, of course, they are *left off 'uns* (left orphans). Skye gives another, but a very good reason, "because their wants are *irreparable*," while P. S. also cleverly suggests, "because they are *in tears*."

No. 10. ST. GEORGE'S CROSS. The *lights* here give "Medical Officer."

```

      A M y

      r E d

    Sun D ays

  O F F I C E R

    suc C our

      m A t

      e L k

```

No. 11. RIDDLE-ME-REE. Everyone discovered Car-a-van. (Caravan.)

No. 12. HISTORICAL CONUNDRUM. John was the king most unfortunate with his laundress, because, you know, he lost all his things in the *Wash !*

No. 13. A MEDICAL MEDLEY. Everyone puzzled, except A. C. K. ! Well, let me explain what Little Mary describes as a "shocking jumble."

| THE MEDLEY. | THE TRANSLATION. |
|---|---|
| Mr. — would falsifying at the extremity of the King of Terrors, his quakers and who which what whom sent for Doctor a let (who) ter to Dr. shortened emperor behold scarlet his donkey is tance. But Doctor what Dr. pauper miss ter — would changed colour. | Mr. Dashwood, lying at the point of death, his *friends* and *relatives* sent for Dr. *Long*, who, *in a letter* to Dr. *Short*, *implored* his *ass*-istance. But what *between* Dr. Long and Dr. Short, *poor* Mr. Dashwood *died.* |

"Shortened emperor behold scarlet"="implored" in this way ; shortened emperor=*imp.* (for Latin *imperator*) behold=lo, and scarlet=red, so we arrive at imp-lo-red, *implored !*

No. 14. A CURIOUS WORD. This is the monosyllable *vague.* Take away the initial letter, and we get the dissyllabic word *ague.*

No. 15. PUZZLE FRENCH SENTENCE. The translation of this is "Un soupir vient souvent d'un souvenir !"

*Seven right*—P.S., A.C.K., Joe, Prospect, Mikado, Tina, Dot; *Six right*—Florrie, Scrooge ; *Five right*—Skye, Isobel, Alsie, Paul Methuen ; *Four right*—Ellie, Little Mary, Bismarck, True Blue, Filomel.

**Letters from Little Folk.**

I was hardly prepared for the little budget of letters which my dear little friends have so kindly sent me—and such excellent little epistles, too ! My first thought was, "Well, I really must set apart a little prize for the letters, independently of the puzzles," and so, as you will have seen above, I have decided to do so. The exigencies of space prevent my printing all, but I have notified below all those which have obtained marks.

**Letter Prize.**

I am sure my little friends will be pleased to hear what Scrooge has to say about her wonderful parrot.

DEAR MR. EDITOR,—I have no brothers or sisters to write about, but I have a parrot which is very amusing, and is very good company when I am alone. It is a grey one with a red tail. Papa brought it from Africa about twenty years ago, and Pollie has become a great favourite. She calls me by my name, and every night she wishes us "Good night." She calls the cat, and then tells him to "Get away." Pollie imitates laughing and sneezing very well indeed. We have taught her to nod her head several times when she receives anything, instead of "thank you." She is very talkative, and makes most absurd speeches when we are talking. If Mamma plays certain pieces of music, Pollie begins to scream and laugh so loudly, that we are obliged to cover her cage with a newspaper. I cannot tell you all the funny things she says and does, as it will take too much room. I like trying to make out your puzzles very much. I made a good many words out in the "Medical Medley," but could not find out all. I am anxious to see the result.—Believe me, dear Editor, your loving friend, SCROOGE (aged 12).

7, Charles Street, Pendleton.

"Mikado," like most little girls, is fond of dicky birds. The following letter will show how kind she is to them :—

DEAR MR. EDITOR,—I hope you like the snow as much as we do. It has been so deep, and it made the trees look lovely. We had to clear a big place in our garden for the dickies ; we called it "the birds' parlour," and we all save something at breakfast and dinner to give to them. I think they are very hungry, for the moment we put it down they come, and very often they quarrel over the biggest pieces. One poor bird has only one leg ; we should like him to get the best.—Yours truly, MIKADO.

12, Upper Tichborne Street, Leicester.

And now a letter from Florrie about her old pussy. I am sure Topsy must be in clover among such kind friends at Dulwich :—

DEAR SIR,—As you have expressed a wish of our writing a little letter to you on any suitable subject, I thought I should like to tell you something of my old cat. Many years ago, when I was *quite a little thing*, two of my brothers brought home a pretty little kitten. I was delighted with it ; it gambolled about, and was up to all kinds of tricks, amusing me for many an hour. Our old servant also had a very great liking for cats and I am afraid partially spoilt her by feeding her at meal-times, and giving her dainties, thus making her gradually dislike her proper food. We never feed her on cat's meat, for some people say it tends to make them rather wild, but usually give her a saucer of milk twice a day, with any bits or scraps that there might be. She is an excellent mouse-catcher, and it was curious to see her teach her kitten the art of mouse-catching. Our old servant left a short time ago, and our pussy did not get over it for a long time ; she showed her sorrow by almost refusing to eat and drink, till she almost looked like a skeleton ; but, however, she has got over it now, and is thriving as well as her old age will permit her. I forgot to tell you that her name is "Topsy." She begs beautifully, and is a very good jumper ; for when I put my hands together, and hold them up a good height, she will jump clean over them. One other thing that she can do (and this she has taught herself) is to open the door with her paw, whenever she wants to go out.—And now, dear Sir, I am, yours truly,

20, St. John's Villas, East Dulwich. FLORRIE.

One mark each : Scrooge, Prospect, Mikado, Skye, Florrie, Ellie, P.S., Little Mary, and Bismarck.

**Notices to Correspondents.**

ELLIE.—Certainly, I shall be very pleased to hear from Percy. You and he may answer as you propose.

PROSPECT.—I am glad you like my puzzles. Many thanks for your efforts to introduce friends.

SKYE.—Your letter about your visit to the Cumberland lead mine was most interesting, but a little too long for publication. What pretty painted flowers in the corner ! Were they yours ?

TIM.—I am shocked to hear you say "the girls have the best chance, as they usually find much more to say than boys." Try.

P.S.—A very nice little letter. Will you please give your name in full next time ?

Answers, having the actual name and address (to which a pseudonym may be added for publication), must have "The Children's" Coupon attached (see advertisement pages), and be addressed to the "PUZZLE EDITOR," THE HOSPITAL, Norfolk House, Norfolk Street, Strand, W.C., not later than Thursday next.

### The In- and Out-Patients' Hospital Diary.

This Diary is so arranged as to make some portion of it appear every week, and the whole in each monthly part. It has been very difficult to compile, and corrections will at all times be gratefully received. It has been suggested that similar information about the larger Provincial and Scotch Hospitals would be useful to many readers. We should be glad to hear if this is felt to be the case.

### ORTHOPÆDIC CASES.

| Hospitals and Terms of Admission. | Physicians, Surgeons, and Medical Officers, with Days and Hours of Attendance. |
|---|---|
| City Orthopædic Hospital, 27, Hatton Garden, E.C. Sec., Mr. E. Dereuth<br>Admission free | *Surgeons*, Mr. E. J. Chance and House-Surgeon. M. Tu. Th. F. 2.30. New cases Tu. F. 2.30 |
| National Orthopædic Hospital for the Deformed, 234, Gt. Portland St., W. Sec., Mr. H. H. Canning<br>Free to poor; others by letter or payment | *In-Physician*, Dr. C. Beevor<br>*In- and Out-Surgeons*, Messrs. F. R. Fisher, M. Th. 2; W. J. Roeckel, Tu. F. 2; E. M. Little, W. 1 |
| Royal Orthopædic Hospital, 297, Oxford Street, W. Sec., Mr. B. Maskell<br>By Governor's letter | *Surgeons*, Messrs. B. E. Brodhurst, H. A. Reeves, C. Read, W. E. Balkwill, H. F. Baker. Daily, 1 |
| St. Bartholomew's Hospital, Smithfield, E.C. | *Surgeon*, Mr. Walsham, M. 2.30 |
| St. George's Hospital, Hyde Park Corner, S.W. | W. 2. *See* General Hospitals |

### PARALYSIS, EPILEPSY, AND DISEASES OF THE NERVOUS SYSTEM GENERALLY.

| | |
|---|---|
| Hospital for Diseases of the Nervous System, 32, Portland Terrace, Regent's Park Road, N.W. Sec., Mr. Howgrave Graham<br>By contributor's letter, or payment according to means, but free to necessitous poor | *In-Physicians*, Drs. J. Althaus, Th.; Hughes Bennett, M.; G. Ogilvie, Tu. Hour 2<br>*Out-Physicians*, Drs. Laycock, F.; F. Hawkins, W.; J. Althaus, Th.; G. Ogilvie, Tu.; H. Bennett, M. Hour, 2<br>*In- and Out-Surgeons*, Messrs. J. Bloxam, A. P. Gould, M. Tu. W. Th. F. 2 |
| National Hospital for the Paralysed and Epileptic, Queen Square, Bloomsbury, W.C. Sec. and Director, Mr. B. B. Rawlings<br>Most of the beds are reserved free to the necessitous poor; the others are for patients who contribute a Guinea a week | *In-Physicians*, Drs. J. S. Ramskill, Tu.; C. B. Radcliffe, M.; J. H. Jackson, F.; T. Buzzard, W. Hour, 2.30<br>*In-Surgeons*, Messrs. W. Adams, M. 3; V. Horsley, F. 2.30<br>*Out-Physicians*, Drs. T. Buzzard, W., H. C. Bastian, Tu.; W. R. Gowers. M.; D. Ferrier, F. 2.30; J. A. Ormerod, Tu. W.; C. E. Beevor, M; F. Hour, 2.30 |
| National Hospital for Diseases of the Heart and Paralysis, 32, Soho Sq., W. Sec., Capt. F. Handley<br>In-patients require recommendation of Life Governor. | *In-Physicians*, Drs. V. Ambler and A. Duncan, M. W. Th.<br>*In-Surgeon*, Mr. G. Bennett, Tu.<br>*Out-Physicians*, Drs. V. Ambler, A. Duncan, R. Varley, J. Murray, M. 2, 7; Tu. 3; W 2, 8; Th. 2; F. 3<br>*Out-Surgeon*, Mr. G. Bennett, Tu. 5 |
| West End Hospital for Diseases of the Nervous System, 73, Welbeck St., W. Hon. Sec., Mr H A. Dowell<br>Children only as in-patients. Out-patients by letter or payment. | *In- and Out-Physicians*, Drs. H. Tibbits, M. Th. 1.30; Stretch-Dowse, Tu. W. 1.30; S. H. Armitage, W. Tu. F. 6<br>*In- and Out-Surgeon*, Mr. Benson |

### DISEASES OF THE SKIN.

| | |
|---|---|
| Charing Cross Hospital, West Strand, W.C. | *Physician*, Dr. Sangster, M. 2 |
| Guy's Hospital, St. Thomas Street, S.E. | *Physicians*, Drs. Pye-Smith, Hale-White, Tu. 12 |
| Hospital for Diseases of the Skin, 52, Stamford St., S.E. Sec., Mr. S. Hayman<br>Free to the necessitous; also by payment | *Physician*, Dr. F. J. Payne<br>*Surgeons*, Messrs. J. Hutchinson, Waren Tay, and Wyndham Cottle. (Out-patients seen M. W. 3 Tu. Th. F. 2) |
| King's College Hospital, Portugal Street, W.C. | *Physician*, Dr. Duffin, F. 1 |
| London Hospital, Whitechapel Road, E. | *Physician*, Dr. S. Mackenzie, Th. 9 |
| Middlesex Hospital, Berners Street, W. | *Physician*, Dr. R. Liveing, Tu. 4 |
| North-West London Hosp. 18, Kentish Town Rd., N.W. | *Physician*, Dr. J. Stowers, Tu. 1.30 |
| Paddington Green Children's Hospital, Paddington | *Physician*, Dr. T. Colcott Fox, S. 9.15 |
| St Bartholomew's Hospital, Smithfield, E.C. | *Surgeon*, Mr. H. Cripps, F. 1.30 |

### DISEASES OF THE SKIN.—*Continued.*

| Hospitals and Terms of Admission. | Physicians, Surgeons, and Medical Officers, with Days and Hours of Attendance. |
|---|---|
| St. George's Hospital. Hyde Park Corner, S.W. | *Physician*, Dr. Cavafy, W. 1.30 |
| St. John's Hospital for Diseases of the Skin, 45, Leicester Sq., W.C. Branch, Markham Square, Chelsea, S.W. Sec., Mr. St. Vincent Mercier. In-patients by letter; out-patients free or by small payment | *Physicians*, Drs. Robinson, Dow, Harries, Campbell, Bourns. Daily 2; M. W. F. 7. Markham Sq. Branch, M. 10 and Th. 10 and 7.<br>*Surgeon*, Mr. J. Milton, Tu. |
| St. Mary's Hospital, Cambridge Place, Paddington, W. | *Surgeon*, Mr. Morris, M. Th. 9.30 |
| St. Thomas's Hospital, Westminster Bridge Road, S.E. | *Physician*, Dr. Payne, W. 1.30 |
| University College Hospital, Gower Street, W.C. | *Physician*, Dr. Crocker, W. 1.30, S. 9.15 |
| Westminster Hospital, Broad Sanctuary, S.W. | *Physician*, Dr. T. C. Fox, W. 1.30 |

### STONE AND OTHER DISEASES OF URINARY ORGANS.

| | |
|---|---|
| London Hospital, Whitechapel Road, E. | Daily, 1 30. *See* General Hospitals |
| St. Peter's Hospital for Stone and Urinary Diseases, Henrietta Street, W.C. Sec., Mr. W. E. Scott.<br>Admission free. Only women and children are seen on Fridays. Operation day, Wednesday | *In-Surgeons*, Messrs. W. J. Coulson, F. R. Heycock.<br>*Out-Surgeons*, Messrs. E. H. Fenwick, M. 2, S. 4 to 7; F. S. Edwards, M. 5 to 7, Th. 2; Heycock, Tu. 2, W. 5 to 7, F. 2 |

### DISEASES AND IRREGULARITIES OF THE TEETH.

| | |
|---|---|
| Belgrave Hosp. for Children, 77 & 79 Gloucester St, Pimlico | *Surgeon*, Mr. G. W. Payne, W. 9 |
| Charing Cross Hospital, West Strand, W C. | *Surgeon*, Mr. Fairbank, M. W. F. 9.0 |
| Dental Hospital of London, Leicester Sq., W.C. Sec., Mr. J. F. Pink<br>Poor free. For special operation a Governor's letter is required | *Surgeons*, Messrs. Canton, Gregson, Hepburn, S. Bennett, Underwood, Woodhouse, Hern, Matheson, Parkinson, Read, Rogers, Truman. Daily, 9 to 11 |
| Evelina Hosp. for Sick Children, Southwark Brdg. Rd. | *Surgeon*, Mr. R. Denison Pedley, Tu. 9 |
| German Hospital, Dalston lane, Dalston, E. | *Surgeons*, Messrs. A. P. Reboul, C. West, Th. 10 to 11 |
| Great Northern Central Hospital, Caledonian Rd, N. | *Surgeon*, Mr. E. Keen, W. 1.30 |
| Guy's Hospital, St. Thomas Street, S.E. | *Surgeons*, Messrs. Moon, Pedley, Tu. Th. F. 12.30 |
| Hospital for Consumption, Brompton, S.W. | *Surgeon*, Mr. C. J. Noble, W., and when wanted |
| Hospital for Sick Children, Gt. Ormond Street, W.C. | *Surgeon*, Mr. Cartwright, W. 9 |
| King's College Hospital, Portugal Street, W.C. | *Surgeon*, Mr. Cartwright, Tu. F. 10 |
| London Hospital, Whitechapel Road, E. | *Surgeon*, Mr. A. W. Barrett, Tu. 9 |
| London Temperance Hosp., Hampstead Road, N.W. | *Surgeon*, Mr. Adolphus Alexander, M. 2 |
| Metropolitan Free Hospital, Gray's Inn Road, W.C. | *Surgeon*, Mr. G. Curnock, Tu. Th. S. 9 |
| Middlesex Hospital, Berners Street, W. | *Surgeons*, Messrs. Bennett, M. 9.30; W. 9; Hern, W. 9, F. 9.30 |
| National Dental Hospital, 149, Gt. Portland Street, W. Sec., Mr. A. G. Klugh.<br>The poor, and all urgent cases admitted free; others by Subscriber's letter | *Surgeons*, Messrs. H. Weiss, W. Weiss, M.; A. Smith, G. Bradshaw, Tu.; G. A. Williams, M. Davis, W.; A. F. Canton, H. G. Read, Th.; T. Gaddes, W. S. Thomson, F.; H. Rose, W. R. Humby, S. Hours 9 to 11 |
| National Orthopædic Hospital, 234, Gt. Portland st, W. | *Surgeon*, Mr. H. Harris |

